# Resolving a century-old enigma: potato ‘Bolters’ originate from instability of the *StCDF1.3* allele

**DOI:** 10.1007/s00122-025-05030-7

**Published:** 2025-09-09

**Authors:** Corentin R. Clot, Ron G. M. van der Hulst, Herman J. van Eck

**Affiliations:** 1 Plant Breeding, Wageningen University & Research, P.O. Box 386, 6700 AJ Wageningen, The Netherlands; 2 The Graduate School Experimental Plant Sciences, Droevendaalsesteeg 1, 6708 PB Wageningen, The Netherlands; 3 Solynta, Dreijenlaan 2, 6703 HA Wageningen, The Netherlands

## Abstract

**Key message:**

**Potato bolters are caused by excision of a transposon from the** ***StCDF1.3*** **allele, resulting in a somatic mutant with late maturity**.

****Abstract**:**

Somatic mutations during vegetative propagation can lead to novel genotypes, known as sports. In cultivated potato (*Solanum tuberosum*), a recurring sport type, called ‘Bolters’, is characterized by vigorous haulms and prolonged flowering. Bolters emerge spontaneously during potato cultivation. While deviating phenotypes are typically rogued during clonal propagation, certain bolters have been selected as sub-clonal strains. Their delayed maturity results in a longer growing season and higher yield, in particular when cultivated under short daylengths. Despite their prevalence and agronomical benefits, the genetic basis of bolters has remained unresolved 160 years after their first description in the literature. We investigated whether allelic variation at the *StCDF1* locus, a central regulator of potato life cycle, underlies the bolter phenotype. We describe 34 bolters from eight cultivars. Bolters are isogenic with their parent varieties and carried new *StCDF1* alleles. These arose from excision events of the Class II TIR transposon disrupting the *StCDF1.3* allele conferring early maturity. Among the newly formed alleles, we predominantly identified *StCDF1.2* variants, characterized by a 7-nucleotide insertion and associated with a mild effect on early maturity. We also found novel variants, including *StCDF1.7*, with a 6-nucleotide in-frame insertion, which appears to confer an even milder shortening of the life cycle. Based on this knowledge, we propose that selecting bolters represents a promising breeding strategy to expand the cultivation range of elite varieties and to enhance allelic diversity at a key regulatory locus.

**Supplementary Information:**

The online version contains supplementary material available at 10.1007/s00122-025-05030-7.

## Introduction

Clonally propagated food crops encompass a diverse group of species, including fruit trees such as apple (*Malus domestica*), as well as root and tuber crops like cassava (*Manihot esculenta*) and potato (*Solanum tuberosum*). Most of these species are heterozygous outbreeders and therefore do not breed true to type through sexual reproduction. In this context, vegetative propagation ensures that their genetic make-up is preserved across generations. Nonetheless, genetic variation can still arise through somatic mutations. These mutations are passed on through cell division, leading to the formation of mosaic individuals (Schoen and Schultz 2019). While mutations in differentiated tissues remain localized, those occurring in meristematic stem cells can be propagated to large sectors of the plant. (Frank and Chitwood 2016). If such sectors are amenable to vegetative propagation, they can give raise to new cultivars. This phenomenon, known as sports, is important in clonal crop breeding, where introducing new traits can be challenging. Sport selection provides new characteristics while retaining the desirable qualities of the parent plant. Several notable sports and their underlying genetic causes were reviewed by Foster and Aranzana (2018). Examples included fruit color mutants in grape (*Vitis vinifera*) (Walker et al. 2006), blood orange (*Citrus sinensis*) (Butelli et al. 2012) and apple (Qian et al. 2014). Other sports affected plant architecture such as the grape periclinal chimera Pinot Meunier, which shows a conversion of tendrils into inflorescences (Walker et al. 2006), or the apple sport “Wijck”, characterized by its transposon induced columnar growth habit (Wolters et al. 2013).

Sports are also relevant in potato breeding, where improvement through so-called strain selection has a long history, particularly in the USA. Notable examples include the selection of the russet-skinned Russet Burbank from Burbank, or the red-skinned Red LaSoda and Norland Dark Red from LaSoda and Norland respectively (Miller 1954). Another type of sport relevant to this study was first described in 1865 by Carrière. He reported plants within the cultivar Marjolin that displayed prolonged vegetative growth and consistently produced flowers and fruits, traits largely absent in the original variety, which rarely flowered. Through strain selection, this variation led to the distinction between the original variety, “Marjolin hâtive”, and the later-flowering sport, “Marjolin tardive”. Similar sports have been repetitively observed and are known by different names across regions: “Bolters” in the UK, “Giant Hills” in the USA, and “Mannetjes” in the Netherlands (Dorst 1924; Murphy and Mckay 1932; Anderson 1936; Heiken 1958). Bolters are characterized by a set of phenotypes, perhaps better described as a syndrome, which typically include a larger, later-senescing canopy with an increased number of inflorescences. Additional traits, such as coarser tubers, slower sprouting and diminished apical dominance of sprouts, longer and thicker stolons, higher anthocyanin production, and enhanced resistance to biotic and abiotic stresses, are also occasionally reported (Dorst 1924; Yarwood 1946; Heiken 1958; Kwiatkowski 1984; Miller et al. 1999; Jansky and Miller 2010). Because of their deviating appearance, bolters are generally rogued out as off-types during seed potato multiplication. Nevertheless, strain selection of bolters within the cultivars Norgold Russet and, more recently, Russet Norkotah has led to the development of cultivars with higher yields at late harvest and better adaptation to Texan growing conditions (Leever et al. 1994; Miller et al. 1999). In 2021, in the USA, two of these strains, Russet Norkotah 278 and Russet Norkotah 296, were grown on more seed potato acreage than the original Russet Norkotah (Jennings 2022).

While the genetic basis of potato bolters remains unknown even in the genomic era (Levy et al. 2018; Pandey et al. 2021), an early hypothesis suggested a linked with the photoperiodic response. This hypothesis was based on the phenotypic similarities between bolters and short-day adapted potatoes grown under long-day conditions (Carson and Howard 1944; Hawkes 1946, 1947). Meanwhile, allelic variants of the gene *StCDF1* have been identified as key contributor to potato adaptation to long-day growing conditions (Kloosterman et al. 2013; Gutaker et al. 2019). The *StCDF1* locus has been repeatedly detected in mapping studies due to its strong influence on maturity traits, including the timing of tuberization and foliage senescence, factors that in turns affect yield, tuber quality, and field resistance to *Phytophtora infestens* and *Verticilium dahliae* (Visker et al. 2003; Tai et al. 2018; Massa et al. 2018; Li et al. 2019; Caraza-Harter and Endelman 2022; Clot et al. 2024). This locus was functionally linked to the gene encoding the transcription factor *StCDF1*, a potato homolog of the *Arabidopsis thaliana* gene *CYCLING DOF FACTOR 1* (Kloosterman et al. 2013). Through repression of *CONSTANS* homologs (*StCOLs*), StCDF1 inhibit the expression of the potato tuberigen *SELF PRUNING 6A* (*StSP6A*)(Abelenda et al. 2016). Under long-day conditions, the wild-type protein StCDF1.1 is subject to ubiquitin-dependent degradation, mediated by the interaction of its C-terminal domains with two proteins: GIGANTEA (StGI) and FLAVIN-BINDING KELCH REPEAT F-BOX PROTEIN 1 (StFKF1). Several allelic variants of *StCDF1* with insertions at the same position in the second exon have been discovered. *StCDF1.3* carries an 865-nucleotide insertion from a transposon, whereas the so-called footprint alleles *StCDF1.2a*, *StCDF1.2b* and *StCDF1.4* each contain different 7-nucleotide insertions (Kloosterman et al. 2013; Gutaker et al. 2019). All these insertions result in premature stop codon, producing C-terminal truncated proteins that evade degradation mediated by StGi and StFKF1.In contrast to plants homozygous for the wild-type *StCDF1.1* alleles, which show late maturity, plants carrying one or more of these mutant alleles exhibit early, day-length-insensitive tuberization and a shortened life cycle. Importantly, the effects of these alleles are not equivalent. Genetic studies have shown that *StCDF1.3* is associated with a most early maturity, followed by the two alleles *StCDF1.2* and *StCDF1.4* characterized by a weaker effect (Hoopes et al. 2022; Caraza-Harter and Endelman 2022; Clot et al. 2024; Ma et al. 2025). A more recently described allele *StCDF1.5* contains a 6-nucleotide in-frame insertion that encodes for a full-length protein associated with a milder acceleration of tuberization compared to the other insertion variants (Ma et al. 2025).

In this study, we tested the hypothesis that bolters could be the result of transposon excision from the *StCDF1.3* allele. Our hypothesis is motivated by the fact that a transposon insertion underlies the dramatic effect of *StCDF1.3* on maturity, that there are many examples of somatic sports caused by transposons activity, and lastly that bolters phenotypically resemble plants carrying *StCDF1* alleles with a attenuated effect on maturity such as the footprint allele *StCDF1.2*.

## Materials and methods

### Collection of plant material and phenotypes

Bolters were sampled in the Netherlands within commercial potato fields planted with the varieties Agata, Agria, Bintje, Eigenheimer, Innovator, Nicola, and Sinora, with the kind permissions of growers. Bolters were recognized from great distance, based on large haulms and profuse flowering relative to the surrounding crop (Supplementary Figure S1). For each cultivar, at least one true-to-type plant was also sampled as control. When putative mosaic plants were detected, bolter and wild-type stems were sampled separately. Additionally, dried leaf samples or DNA from Russet Norkotah and five sub-strain selections (selection 3, selection 8, strain 112, strain 223, strain 278, and strain 296) were kindly provided by Dr. Isabel Vales (Texas A&M University), Dr. Caroline Gray (Colorado State University), and Dr. Mariëlle Muskens (Agrico Research). All samples information including year of collection, field location, and provider’s names are available in Supplementary Table S1.

In many instances bolters were opportunistically collected. As a result, phenotypical descriptions are lacking for most samples. Here we measured stem length and counted the number of flowering and senescent inflorescences of the bolters of cultivar Nicola (Supplementary Table S2) to highlight the key characteristic of the bolter syndrome. Data were visualized using the R package ggplot2 (Wickham 2016), and group means were compared using a t test implemented via the stat_compare_means() function from the R package ggpubr (Kassambara 2023).

### Genotyping

For all samples collected in the Netherlands, DNA was isolated from leaf punches by LGC, Berlin, Germany and subsequently analyzed by LGC’s genetic platform ‘SeqSNP’ which is a single primer enrichment sequencing technology (SPET) (Barchi et al. 2019). We essentially followed the procedure described in Schilling et al. (2024) but instead used Solynta’s genome-wide set of 790 proprietary probes (undisclosed data). Importantly, one of these probes overlaps with the gene *StCDF1* (*Soltu.DM.05G005140*). This probe targets a 150-nucleotide region located downstream of position 4,487,957 on chromosome 5 in DMv6.1 reference genome.

For the Russet Norkotah samples, DNA was either resuspended in distilled water or directly isolated from dried leaves using the DNeasy 96 Plant Kit (QIAGEN), following the manufacturer’s instructions. PCR was performed with PhusionTM High-Fidelity DNA polymerase (Thermo Scientific). The sense 5’-CGAAGAATGCCTGCAATCGG and antisense primer 5’-ACAAGGCTGCTGGATTAGCTT were designed using Primer3Plus http://www.bioinformatics.nl/primer3plus/, and cycling conditions (30x) were as follows: initial denaturation at 95 °C 4 min, 30 cycles at 95 °C, 56 °C and 72 °C for, 30 s, 1 min and 2 min, resp. in reactions of 50 μL total volume. This PCR amplified the variable region of *StCDF1* including the putative transposon insertion site. The PCR products were visualized on 1% agarose gel. Prior to sequencing the PCR products were cleaned using the DNA Clean & Concentrator-5 kit (Zymo Research), following the manufacturer’s instructions. Premium PCR Sequencing of the resulting products was performed by Plasmidsaurus Inc., USA, using Oxford Nanopore Technology (ONT), and 5,000 reads were obtained per sample.

### To distinguish admixture from true bolters

SeqSNP sequencing data were aligned to their corresponding amplicon target regions on the reference genome DMv6.1 (Pham et al. 2020) using minimap2 v2.24 (Li 2018). For each amplicon, genetic variation among mapped reads was used to group reads into haplotypes through a haplotype-based clustering approach implemented in QualitySNPng rev39 (Nijveen et al. 2013). These haplotype groups were then compared against Solynta’s allele database. For each of the 790 loci, up to four alleles were called and encoded by a unique identifier separated by an underscore (Supplementary Table S3). The genetic distance between samples was estimated by counting the number of genome-wide allelic mismatches between every pair of samples with a custom R script. The resulting mismatch matrix was visualized with the R package pheatmap (Kolde 2019) with samples clustered by similarity using the hclust() function and the complete linkage method from the R package stats (R Core Team 2013). Candidate bolters failing to cluster with their respective varieties were classified as the result of admixture and discarded from further analysis (Supplementary Figure S2).

### Discovery, frequency and translation of CDF1 alleles

For the SNPseq genotyped samples, reads aligned to the *StCDF1* locus were grouped into alleles, excluding read groups with a frequency below 5%. To facilitate visualization, reads corresponding to *StCDF1.1* (the wild-type alleles, lacking indels in the second exon) were grouped under the generic name *StCDF1.1*. Similarly, all clipped reads due to the presence of a transposon sequence were annotated as *StCDF1.3*. The remaining read groups corresponded to previously described footprint alleles *StCDF1.2a*, *StCDF1.2b*, and *StCDF1.4* (Kloosterman et al. 2013; Gutaker et al. 2019; Ma et al. 2025), and two alleles *StCDF1.7* and *StCDF1.8* firstly described in this study.

For Russet Norkotah samples, ONT reads were aligned to the genomic sequence of *Soltu.DM.05G005140* in Geneious using minimap2 with default parameters (-x map-ont –frag = yes –secondary = yes -N 5 -p 0.8). Variant discovery was performed using Geneious’s Find Variants tool with a minimum variant frequency of 5%. Subsequently, reads uniquely matching to the different *StCDF1* alleles identified were counted using seqkit grep and the patterns presented in Table 1. For all samples read counts and allele frequencies are shown in Supplementary Table S4. These frequencies were visualized as a heatmap using the R package pheatmap (Kolde 2019). Finally, the genomic sequences of all alleles were in silico translated using Geneious.

**Table 1:** *StCDF1* allele specific patterns use to count reads uniquely matching each allele using seqkit grep. For *StCDF1.3*, one pattern was used for each side of the transposon insertion. The different insertions relative to *StCDF1.1* are indicated in bold

Alleles	Grep patterns
*StCDF1.1*	CTAAAAGCTCTATATGGTCAACACTAGGTATCAGGAATGAGAAGATTGA
*StCDF1.2a*	CTAAAAGCTCTATATGGTCAACACTAG**CCACTAG**GTATCAGGAATGAGAAGATTGA
*StCDF1.2b*	CTAAAAGCTCTATATGGTCAACACTAG**TCACTAG**GTATCAGGAATGAGAAGATTGA
*StCDF1.7*	CTAAAAGCTCTATATGGTCAACACTAG**CACTAG**GTATCAGGAATGAGAAGATTGA
*StCDF1.3*	CTAAAAGCTCTATATGGTCAACACTAG**GTAAGGCTGGGCACCGGACCGGAATGG**
*StCDF1.3*	**ACCCGTTCCGGTGCCCAGCCTTAACACTAG**GTATCAGGAATGAGAAGATTGA

## Results

### Bolters are isogenic but show a distinct phenotype

**Fig. 1:**
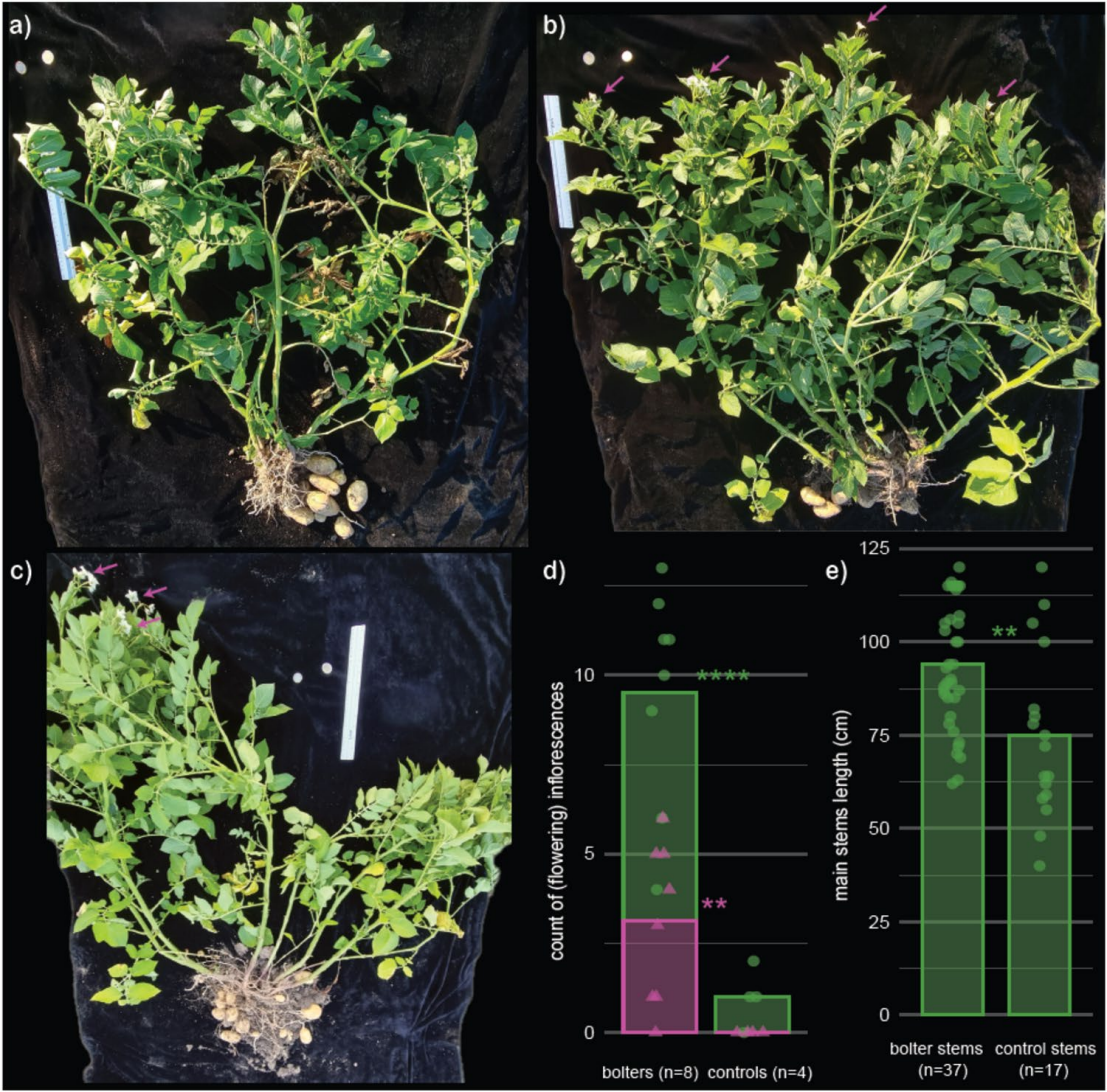
Photographs of Nicola control (**a**), bolter (**b**), and mosaic (**c**) clones. A 30 cm ruler is included in each image for scale. Pink arrows indicate flowering inflorescences. **d** Number of flowering (pink triangles) and total (green circles) inflorescences of Nicolas bolters and controls. **e** Main stem length of Nicolas bolters and controls. Statistical significance from t tests is indicated as follows: ** for *P* < 0.01 and **** for *P* < 0.0001

Over three cropping seasons between 2020 and 2024, 31 candidate bolters were identified in ware potato fields of different varieties: 5 in Agata, 3 in Agria, 3 in Bintje, 3 in Eigenheimer, 2 in Innovator, 13 in Nicola, and 2 in Sinora. These clones stood out from the surrounding crop due to their larger haulms, delayed foliage senescence, and profuse flowering (Supplementary Figure S1). Four out of the thirteen Nicola bolters exhibited a mosaic phenotype, with some stems displaying typical bolter characteristics while others appeared true-to-type (Fig. 1c). Phenotypic measurements were taken from eight Nicola bolters (excluding mosaic individuals) and four true-to-type control plants to quantitatively assess bolter characteristics. Bolters exhibited a significantly higher number of inflorescences compared to controls, with a mean of 9.5 versus 1.0, respectively. More strikingly, bolters were still flowering with on average 3.5 flowering inflorescences, while all inflorescences on control plants had senesced by the time of sampling (Fig. 1d). In addition, bolters had significantly longer main stems, with an average length of 93.9 cm compared to 74.8 cm in control plants. Qualitative observations also confirmed that foliar senescence was more advanced in control plants (Fig. 1a) than in bolters (Fig. 1b). Collectively, these observations suggest an overall later maturity status of bolters compared with controls.

To ensure that these phenotypic differences were not due to contamination (e.g., volunteer plants or mixed-up seed tubers), we genotyped both bolter and control plants using SeqSNP across 790 loci. The resulting dataset (Supplementary Table S3) was used to calculate a pairwise mismatch matrix (i.e., number of differing alleles), which served as a proxy for genetic distance to confirm whether bolters were isogenic with control plants. Indeed, most bolters were isogenic with the control plants, with the exception of three plants Agria-B1, Agria-B2, and Nicola-B5, which appeared to be contaminants and were excluded from subsequent analyses (Supplementary Figure S2). Although these contaminants originated from two different fields, they were genetically identical to each other and related to Agria.

After this curation step, the dataset included 9 control, 24 bolter, and 4 mosaic clones from seven different varieties. Our collection was supplemented with DNA from Russet Norkotah and six sub-strains from Russet Norkotah (strains 112, 223, 278, 296, and selections 3 and 8) derived from strain selection programs in the USA.

### *StCDF1* allelic variation in potato bolters

**Fig. 2:**
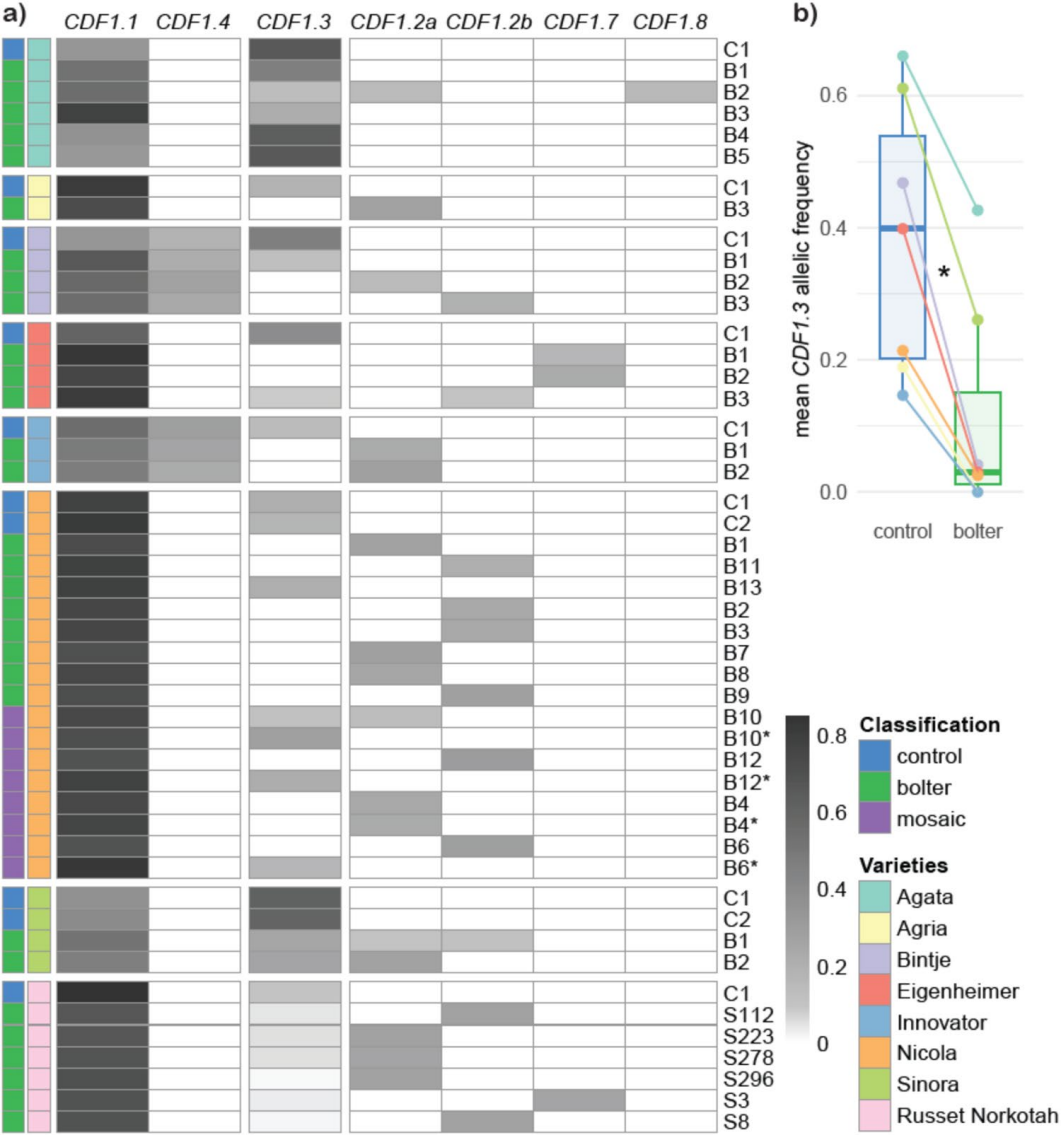
**a** Heatmap showing the relative read depth of different *StCDF1* alleles. Each row represents an individual with a first color code to indicate controls (blue), bolters (green), and mosaic plants (purple) and a second color code for the eight potato varieties: Agata (light teal), Agria (pale yellow), Bintje (lavender), Eigenheimer (salmon pink), Innovator (sky blue), Nicola (soft orange), Sinora (light green), and Russet Norkotah (pale pink). Additional rows corresponding to wild-type stems from mosaic clones are marked by an asterisk*.* **b** Boxplots showing the relative frequency of the *CDF1.3* reads in control and bolter samples for each variety. Statistical significance from t test is indicated by an asterisk (P < 0.05).

**Fig. 3:**
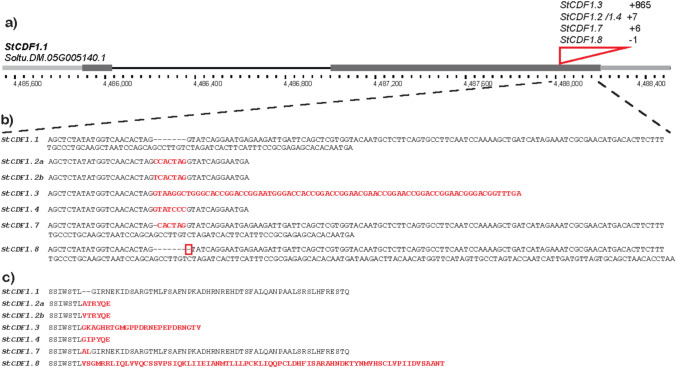
**a** Gene model of *StCDF1.1* showing its physical position on the DMv6.1 reference genome, with the variable region in the second exon highlighted. **b** Nucleotide sequence alignment of the different *StCDF1* alleles identified in this study at the variable region. **c** Predicted amino acid sequence alignment of the different alleles in the variable region.

Next, we focused on our candidate gene *StCDF1*, which has been shown to be the major genetic determinant of maturity variation in cultivated potato (Kloosterman et al. 2013; Caraza-Harter and Endelman 2022; Ma et al. 2025). We assessed the allelic composition at the *StCDF1* locus using SeqSNP short-read data for all varieties, except for Russet Norkotah control and strains, for which the variable region of *StCDF1* was sequenced using ONT reads. Strikingly, all varieties carry a combination of the late-maturity allele *StCDF1.1* and the early-maturity allele *StCDF1.3*. In addition, Bintje and Innovator also possess the *StCDF1.4* allele. The earliest maturing varieties, Agata and Sinora, appear to be duplex for *StCDF1.3* (Fig. 2). This allele presents a transposon insertion in the second exon, which leads to a truncated version of the StCDF1 protein (Fig. 3). When comparing *StCDF1* allele read counts between control plants and bolters across varieties, we observe a significant reduction in *StCDF1.3* reads in bolters. In the early-maturing, *StCDF1.3*-duplex varieties Agata and Sinora, bolters show an approximately 50% decrease in *StCDF1.3* read counts. In contrast, in simplex varieties, *StCDF1.3* is entirely absent in 17 bolters, with no supporting reads detected (Fig. 2). In 28 bolters (all but six), we identified new *StCDF1* alleles, absent in the original variety. These new alleles appear to result from the excision of the transposable element from *StCDF1.3* (*StCDF1.3-TE*) letting behind a “footprint” in the form of different indels. Among these footprint alleles, the most common were *StCDF1.2a* (15 occurrences) and *StCDF1.2b* (11 occurrences), both containing a 7-nucleotide insertion. These *StCDF1.2* alleles have been previously identified in cultivated potato germplasm and are also associated with early maturity, though their effect is milder than that of *StCDF1.3* (Kloosterman et al. 2013; Caraza-Harter and Endelman 2022). In addition, two novel alleles were discovered: *StCDF1.7* (3 occurrences), which harbors a 6-nucleotide in-frame insertion and is predicted to encode a full-length protein with two additional amino acids, and *StCDF1.8*, which carries a 1-nucleotide deletion predicted to produce a protein with a longer and altered C-terminal region (Fig. 3).

Three of the four Nicola bolters, classified as mosaic during field sampling, were confirmed to be chimeras. In these plants, some stems carry the ancestral *StCDF1.3* allele and other stems harbor the new alleles *StCDF1.2a* (Nicola-B10) or *StCDF1.2b* (Nicola-B6 and B12). In contrast, Nicola-B4 appeared to be a non-mosaic bolter, with both stem samples bearing *StCDF1.2a* and lacking *StCDF1.3*. Interestingly, the bolter stem of the mosaic clone Nicola-B10, unlike the other Nicola bolters, still harbored *StCDF1.3* alongside the new allele *StCDF1.2a*. This suggests the presence of two genetically different cell linages in the sampled tissue, potentially indicating periclinal chimerism. The same phenomenon could explain the presence of both *StCDF1.2a* and *StCDF1.8* in Agata-B2, as well as the co-occurrence of *StCDF1.2a* and *StCDF1.2b* in Sinora-B1.

Upon re-examining the *StCDF1* sequence reads from the six bolters in which no apparent new alleles were initially detected, notable variation was identified in four cases. In Agata-B1 and Bintje-B1, novel variants of *StCDF1.3* were observed, supported by 57 reads. These *StCDF1.3*-like haplotypes still have a transposon like insert but also harbor several SNPs and are predicted to encode unique StCDF1 proteins (Supplementary Figure S3). Unfortunate, the read length of the SeqSNP platform was insufficient for full characterization of these alleles. In Agata-B1 and B4, the footprint allele *StCDF1.2a* was detected at low frequency, supported by 7 and 17 reads, respectively, but was initially filtered out due to this low representation. Similarly, in Agata-B3, 11 reads supported the presence of the allele *StCDF1.2b*. Given the previous identification of mosaic individuals, we cannot exclude the possibility that these clones are periclinal chimeras, with the *StCDF1.2* alleles confined to a single cell layer and therefore underrepresented in the sequencing data.

Russet Norkotah and its strains were analyzed using a different molecular assay, but similar changes were observed at the *StCDF1* locus. Russet Norkotah is known to carry two *StCDF1.1* alleles and two *StCDF1.3* alleles. However, due to the shorter length of *StCDF1.1,* this allele amplifies more efficiently during PCR, resulting in an approximately eightfold lower read depth for *StCDF1.3* (Supplementary Table S4). In all strains, sequence reads corresponding to both *StCDF1.1* and *StCDF1.3* were detected, along with newly emerged footprint alleles showing higher read depth, likely due to improved PCR efficiency. This pattern suggests that transposon excision occurred in one of the *StCDF1.3* alleles, converting the genotype from duplex to simplex. Russet Norkotah strains 223, 278 and 296 carry a new *StCDF1.2a* allele, while strain 112 and selection 8 carry a new *StCDF1.2b* allele. In contrast, selection 3 carries the rarer *StCDF1.7* allele, predicted to encode for a full-length *StCDF1* protein, potentially conferring a later maturity than the *StCDF1.2* alleles found in other strains.

Overall, the observed pattern strongly suggests that bolters are characterized by a change in their allelic composition at the *StCDF1* locus, driven by the excision of *StCDF1.3-TE*, producing footprint alleles causing later maturity.

## Discussion

### Recurrent emergence of novel StCDF1 alleles and loss of StCDF1.3 in bolters

Our results provide unambiguous evidence that, across the eight potato varieties, 28 out of 34 bolters carry *StCDF1* alleles absent in their respective controls, along with a clear loss or strong reduction in *StCDF1.3* read frequencies. This robustly supports our initial hypothesis that the occurrence of bolters in potato is primarily driven by genetic instability of the *StCDF1.3* allele. In a few cases, the data were less conclusive. Two clones exhibited novel alleles at frequencies below 5%, which we interpret as potential periclinal chimeras. However, this interpretation remains tentative and requires further empirical validation, which is discussed in the next section. Another two clones carried *StCDF1.3* variants predicted to encode altered proteins, but due to technical limitations of our SPET genotyping platform, we were unable to fully characterize these alleles. More challenging for our hypothesis were two clones (Agata-B5 and Nicola-B13) that showed no detectable new *StCDF1* alleles. These cases could be due to human error (mislabeling or phenotyping mistakes), or to somatic mutations occurring outside of the sequenced region of *StCDF1*, or even in other genes involved in photoperiod response. Despite these exceptions, the consistency of our findings across multiple bolters and multiple varieties supports a direct link between *StCDF1.3-TE* excision and the emergence of bolters. This mechanism is consistent with the classification of the *StCDF1.3-TE* sequence as a “cut-and-paste” Class II TIR transposon in the RepetDB database (Amselem et al. 2019). Interestingly, the repeated detection of de novo* StCDF1.2* alleles in bolters suggest that these alleles already frequently observed in the potato germplasm (Kloosterman et al. 2013; Gutaker et al. 2019; Ma et al. 2025), descend from *StCDF1.3*. Moreover, the bias toward *StCDF1.2a*, and to a lesser extent *StCDF1.2b*, footprints following *StCDF1.3-TE* excision points to non-random excision outcomes.

### Layer specificity and frequency of StCDF1 mutations

A major question stemming from our findings concerns the layer specificity of the observed mutations at the *StCDF1* locus. Plant meristems are typically organized into three distinct layers that largely remain isolated during organ development. For instance, the epidermis and trichomes arises from L1, the leaf mesophyll, leaf veins and gametes from L2, and the vascular system from L3. While the separation between L1 and L2 is generally maintained, studies in potato and *Arabidopsis thaliana* have shown that in axillary meristems, L2 cells can occasionally invade the L3 layer (Howard 1972; Jenik and Irish 2000; Amundson et al. 2023). In his MSc thesis, Kwiatkowski (1984) tested an earlier hypothesis by Howard (1968) proposing that bolters originate as periclinal chimeras, with the initial mutation arising in L2 and could later spread to L3. Kwiatkowski's observations from tissue culture regenerants supported this idea. He found that the severity of the bolter phenotype correlated with the tissue layers affected: mutations limited to L2 produced mild bolters (“semi-giants”), while those also present in L3 resulted in more extreme forms (“super-giants”). While Kwiatkowski regenerated plants from shoot tips, shoot meristems, and different tuber tissues, recent work by Amundson et al. (2023) demonstrated that regeneration from potato leaf protoplasts can produce genetically uniform plants from all layers of periclinal chimeras. This strategy could be used to test the layer specificity of *StCDF1* mutations in bolters and to generate genetically uniform, non-mosaic individuals. However, caution is warranted, as protoplast regeneration in potato is known to induce extensive genomic instability, which will introduce additional variation (Fossi et al. 2019).

Another key question concerns the frequency at which bolters emerge, which can now be reframed as the frequency of *StCDF1.3-TE* excision. From the bolter literature (Stanton 1952; Heiken 1958), we know that the strongest predictor of bolter emergence is the variety of origin. This can now be more precisely explained by the presence of at least one *StCDF1.3* allele within such variety. It is also well established that the frequency of somatic mutations is layer-specific, with L1 accumulating more mutations than L2 or L3 (Amundson et al. 2023; Goel et al. 2024). Transposable element activity is both environmentally responsive and epigenetically regulated (Fedoroff 2012). A variety of stresses are known to activate transposable elements, notably retrotransposons (Grandbastien 1998). Among these, tissue culture-induced epigenetic reprogramming is recognized as an inducer of transposable elements activity (Kaeppler et al. 2000). In potato, in vitro propagation history has been associated with elevated levels of transposable elements in the genome (Bozan et al. 2023). This points to tissue culture as a potential method for generating bolters and echoes the well-documented case of skin color reversion in potato (Momose et al. 2010). In a red-skinned clone, a gene controlling purple pigmentation was inactivated by insertion of a miniature inverted-repeat transposable element (MITE). In a purple somaclonal variant regenerated from protoplasts, the MITE had excised, restoring gene function and purple color. This example further supports the idea that transposons, far from being inert, can be reactivated by tissue culture and cause phenotypic changes of potential agroeconomic interest.

### The breeding potential of bolters

In the USA, strain selection of bolters has led to the development of new cultivars, some of which have outperformed in yield and acreage their parent varieties, such as some of the Russet Norkotah strains included in this study (Leever et al. 1994; Miller et al. 1999; Jennings 2022). The strain selection process typically begins with the identification of bolters through vine selection, followed by multiple years of selection for agronomic traits such as tuber yield and quality (Miller et al. 1999). When evaluating yield potential, the duration of the growing season and timing of harvest is critically important. While all Russet Norkotah strains examined in this study produced higher yields than the original Russet Norkotah at late harvest (150 days after planting), this was not the case at early harvest (105 days after planting) for selection 3, which yielded close to 50% less (Spear et al. 2017). In this manuscript, we reveal a molecular mechanism that helps explain the distinct agronomic performance of selection 3. Specifically, this strain carries a unique allele, *StCDF1.7*, characterized by a six-nucleotide in-frame insertion that results in a full-length protein. Aside from a single SNP causing an amino acid substitution, *StCDF1.7* is otherwise identical to the recently described *StCDF1.5* (Ma et al. 2025). This allele leads to delay tuberization by approximately 20 days compared to *StCDF1.3*. In contrast, the *StCDF1.2* alleles present in the other Russet Norkotah strains were associated with a shorter delay of around 10 days. Therefore, we postulate that *StCDF1.7* delays tuberization in selection 3 compared to other Russet Norkotah strains, resulting in lower yield at early harvest. The interplay between growing season length, *StCDF1* allelic composition, and yield highlights that the success of bolter-derived strains depends on their interaction with both environmental conditions and crop management practices. Ma et al. (2025) hypothesized that *StCDF1.3*, and to a lesser extent *StCDF1.2*, confers strong adaptation to long-day conditions, whereas *StCDF1.1* and *StCDF1.5* may be more suited to low-latitude environments. Building on this, strain selection of bolters within successful *StCDF1.3*-bearing cultivars could offer a strategy to expand their cultivation range to subtropical regions with limited photoperiod variation and longer growing seasons such as southern China, the southern USA, or the Maghreb.

The bolter phenotype, historically described as a syndrome involving multiple traits (Heiken 1958), has been shown to differ in strength depending on whether mutations are confined to specific layers or present throughout the plant (Kwiatkowski 1984). Our findings add a new dimension to this variability by linking phenotypic differences among bolters to allelic diversity at the *StCDF1* locus. From a breeding perspective, bolters represent an opportunity for Eco-TILLING (Comai et al. 2004). The spontaneous *StCDF1.3-TE* excisions can generate novel alleles absent from current germplasm, providing valuable genetic variation for breeding. In our experience, bolters were readily identified when sampling *StCDF1.3*-bearing cultivars at the early senescence stage BBCH 901 (Kacheyo et al. 2021). Using PCR-based fragment analysis combined with ONT sequencing, bolter screening becomes a practical approach to discover new *StCDF1* alleles, assess their tissue-specific distribution, and evaluate their impact on the diverse traits associated with the bolter phenotype. Further expression profiling of distinct bolters within a cultivar could clarify the regulatory mechanisms underlying traits variation, both among bolters and in comparison to their non-bolter counterparts (Levy et al. 2018). Collectively, our findings reframe bolters not as undesirable off-types, but as a resource for expanding the agroecological range of successful cultivars, enriching allelic diversity at a key locus, and deepening our understanding of *StCDF1* function.

## Supplementary Information

Below is the link to the electronic supplementary material.

Supplementary file1 (PDF 9213 kb)

Supplementary file2 (XLSX 210 kb)

## Data Availability

All data, including ONT reads for Russet Norkotah and its strains, as well as the code used to reproduce the results and figures in this article, are temporary available at: https://figshare.com/s/48bd85349723e09c5b96 (a permanent DOI will be created for the published version of this manuscript).

## Availability of material

Collected bolters can be shared for the duration that they are being maintained upon reasonable request. The plants are without plant passport or health certificate which may hinder exchange.
