# Supplementary material for: Resolving a century-old enigma: potato ‘Bolters’ originate from instability of the StCDF1.3 allele
Source: Theor Appl Genet. 2025 Sep 9;138(10):245. doi: 10.1007/s00122-025-05030-7 (PMC12420743; doi:10.1007/s00122-025-05030-7)
Supplement: Supplementary file 1 — Supplementary file1 (PDF 9213 kb) [file 122_2025_5030_MOESM1_ESM.pdf]

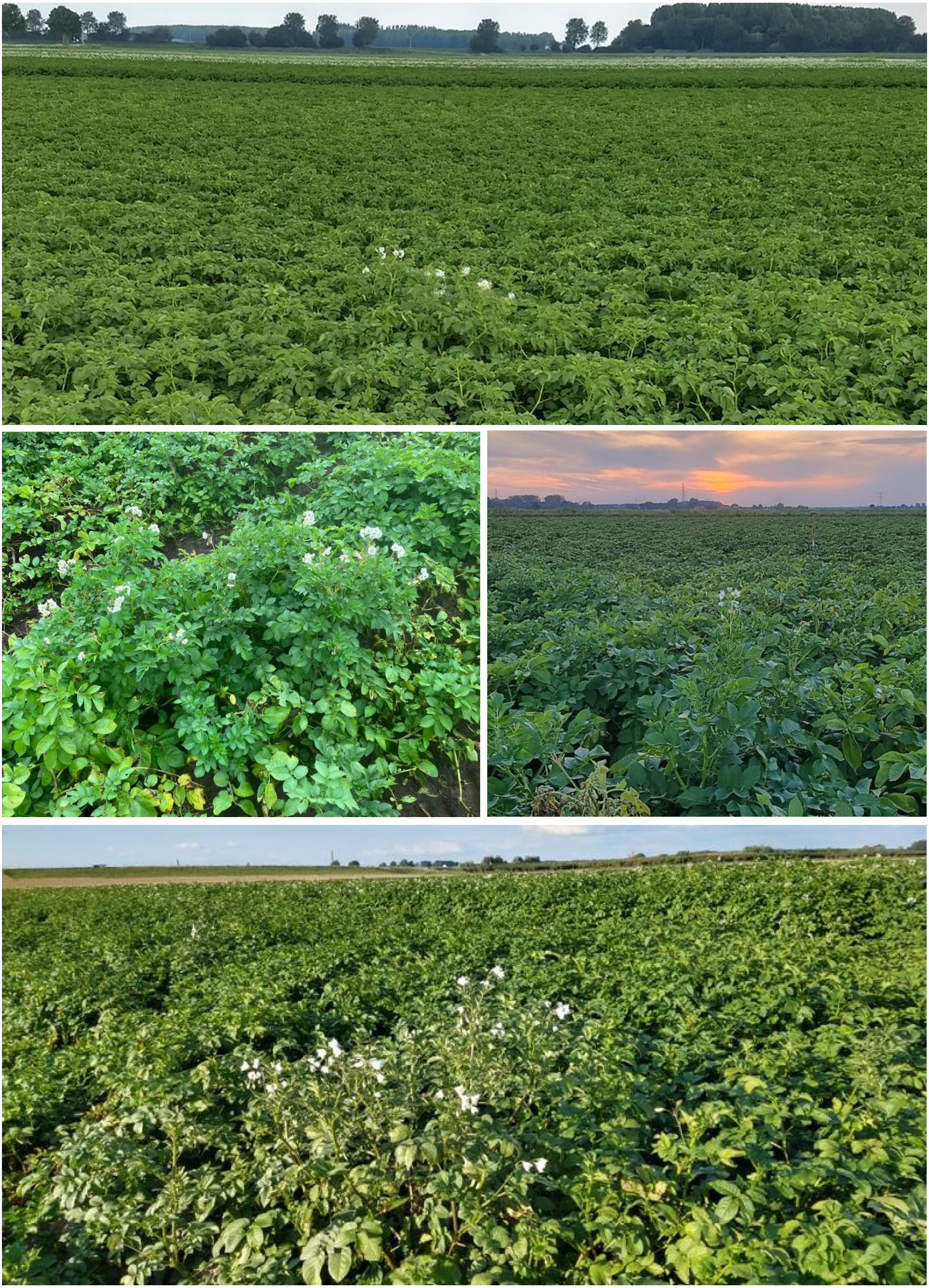

Supplementary Figure S1: Field view of Agata (**a**), Sinora (**b**), Nicola (**c**) and Eigenheimer (**d**) bolters.

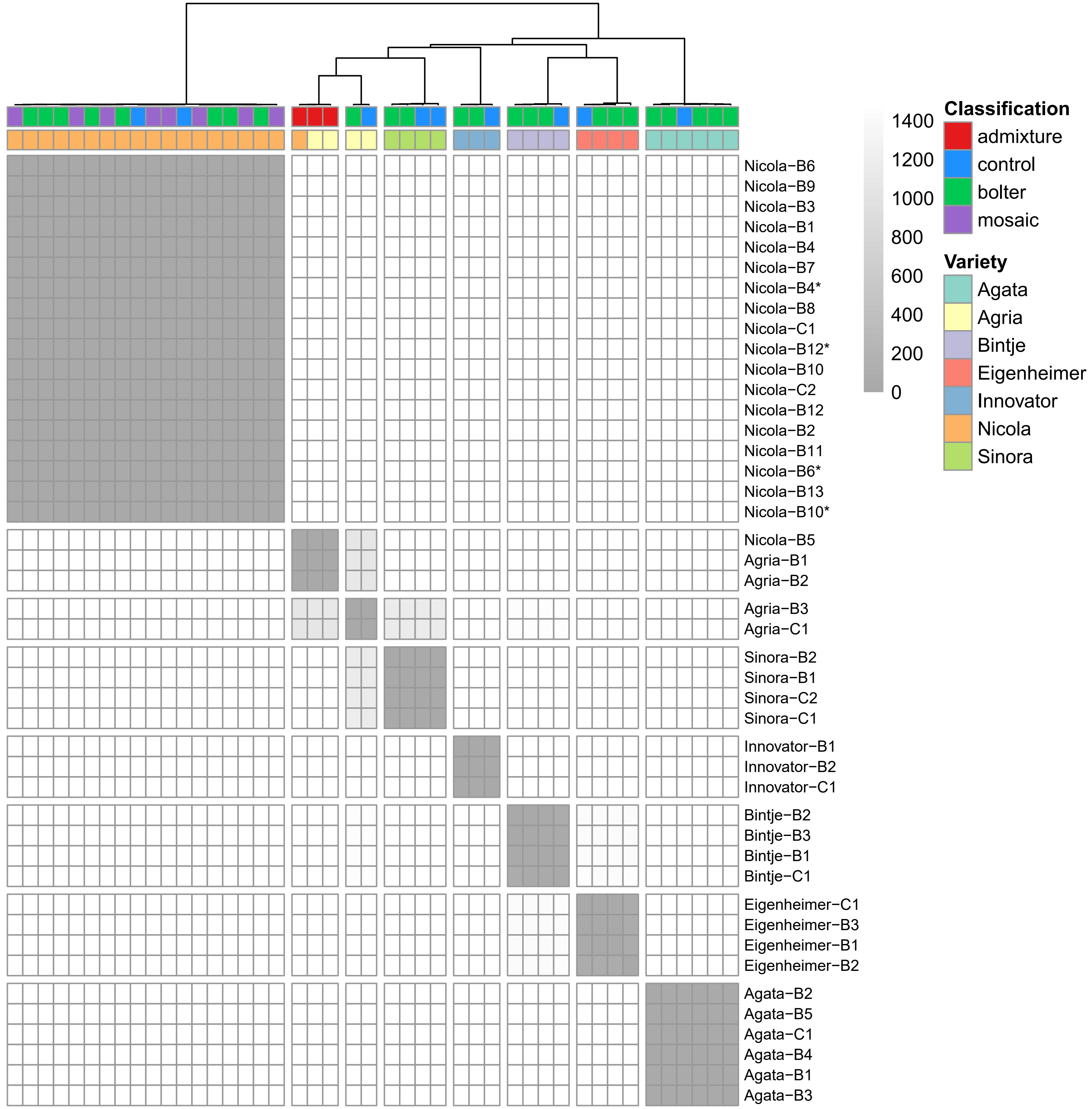

Supplementary Figure S2: Heatmap showing the number of SeqSNP allelic mismatches between control (blue), bolter (green), and mosaic (purple) clones across seven potato varieties: Agata (light teal), Agria (pale yellow), Bintje (lavender), Eigenheimer (salmon pink), Innovator (sky blue), Nicola (soft orange), and Sinora (light green). Clustering based on genetic similarity highlights a distinct group of admixture plants (red). Rows representing wild-type stems from mosaic clones are marked with an asterisk.

a)

>*StCDF1.3*
TAATTTGCAGAATAGAGAAGGCGAGAGATGTGTACTGATTCCAAAGACATTAAGGATTCA 60
>*StCDF1.3*_Agata_B1
TAATTTGCAGAATAGAGAAGGCGAGAGATGTGTACTGATTCCAAAGACATTAAGGATTCA 60
>*StCDF1.3*_Binje_B1
TAATTTGCAGAATAGAGAAGGCGAGAGATGTGTACTGATTCCAAAGACATTAAGGATTCA 60

>*StCDF1.3*
TGATCCAAATGAAGCGGCTAAAAGCTCTATATGGTCAACACTAG**GTAAGGCTGGGCACCG** 120
>*StCDF1.3*_Agata_B1
TGATCCAAATGAAGCGGCTAAAAGCTCTATATGGTCAACACTAG**GTAAGGCTGGGCACCG** 120
>*StCDF1.3*_Binje_B1
TGATCCAAATGAAGCGGCTAAAAGCTCTATATGGTCAACACTAG--**CAGGCTGGGCACCG** 118

>*StCDF1.3*
GACCGGAATGGGACCACCGGACCGGAACGA-- 150
>*StCDF1.3*_Agata_B1
GACCGGAATGGGACCACGGACAAGGATCTA-- 150
>*StCDF1.3*_Binje_B1
GACCGGAATGGGACCACCGGACCGGAACGAAC 150

b)

>CDF1.3
NLQNREGERC VLIPKTLRIH DPNEAAKSSI WSTL**GKAGHR TGMGPPDRN** 49

>CDF1.3_Agata_B1
NLQNREGERC VLIPKTLRIH DPNEAAKSSI WSTL**GKAGHR TGMGPRTRI** 49

>CDF1.3_Binje_B1
NLQNREGERC VLIPKTLRIH DPNEAAKSSI WSTL**AGWAPD RNGTTGPER** 49

Supplementary Figure S3: **a)** Nucleotide sequence alignment of the SeqSNP-amplified region of the canonical *StCDF1.3* allele and the variants identified in Agata-B1 and Bintje-B1 **b)** Predicted amino acid sequence alignment corresponding to the nucleotide sequences shown above. In both alignments, the transposon region is highlighted in bold and deviation from the canonical *StCDF1.3* are highlighted in red.
